# Alcohol and the Lung

**DOI:** 10.35946/arcr.v38.2.07

**Published:** 2017

**Authors:** Ashish J. Mehta, David M. Guidot

**Affiliations:** Ashish J. Mehta, M.D., is an Assistant Professor of Medicine in the Department of Medicine, Division of Pulmonary, Allergy, Critical Care & Sleep Medicine, Emory University, Atlanta, Georgia, and a Staff Physician at the Atlanta VA Medical Center, Decatur, Georgia. David M. Guidot, M.D., is a Professor of Medicine in the Department of Medicine, Division of Pulmonary, Allergy, Critical Care & Sleep Medicine, Emory University, Atlanta, Georgia, and a Staff Physician at the Atlanta VA Medical Center, Decatur, Georgia

**Keywords:** Alcohol consumption, alcohol use disorder, alcoholic lung, lung, lung disease, lung injury, respiratory system, pulmonary system, alveolar macrophage, antioxidant

## Abstract

Among the many organ systems affected by harmful alcohol use, the lungs are particularly susceptible to infections and injury. The mechanisms responsible for rendering people with alcohol use disorder (AUD) vulnerable to lung damage include alterations in host defenses of the upper and lower airways, disruption of alveolar epithelial barrier integrity, and alveolar macrophage immune dysfunction. Collectively, these derangements encompass what has been termed the “alcoholic lung” phenotype. Alcohol-related reductions in antioxidant levels also may contribute to lung disease in people with underlying AUD. In addition, researchers have identified several regulatory molecules that may play crucial roles in the alcohol-induced disease processes. Although there currently are no approved therapies to combat the detrimental effects of chronic alcohol consumption on the respiratory system, these molecules may be potential therapeutic targets to guide future investigation.

Few social practices have had a longer or more complicated history in human civilization than the consumption of alcohol. As documented in academic writings, but even more commonly in art and music, humans have consumed alcohol for thousands of years, and drinking is either a celebrated facet of social activities or a proscribed practice, depending on the local moral or religious views. Although alcohol intoxication has been described in various written recordings since antiquity, it is only relatively recently that its true effects on lung health have been recognized. In the latter years of the 18th century, the first Surgeon General of the United States of America, Benjamin Rush (for whom the medical school in Chicago is named), noted that excessive alcohol consumption was associated with pneumonia (see Happel and Nelson 2005; Mehta and Guidot 2012). More than a century later, William Osler wrote that alcohol abuse was the most important risk factor for pneumonia (see Happel and Nelson 2005; Mehta and Guidot 2012). As modern medicine evolved throughout the 20th century, it became abundantly clear that alcohol use disorder (AUD) rendered people more susceptible to a wide variety of lung infections, including bacterial pneumonias and tuberculosis, and increased morbidity and mortality. In a now-classic modern citation, Perlino and Rimland (1985) coined the term “alcoholic leukopenic pneumococcal sepsis syndrome” when they published a case series of patients with underlying AUD who suffered from pneumococcal pneumonia and sepsis associated with leukopenia that was associated with a mortality of more than 80 percent. Excessive alcohol consumption seems to increase susceptibility to pneumonia through multiple mechanisms. The major factors include an increased risk of aspiration, abnormalities in the way particles are eliminated from the conducting airways through the mucus (i.e., in mucociliary clearance), and impaired activity of one branch of the immune system (i.e., innate immunity) within the lower airways (for reviews, see Joshi and Guidot 2007; Mehta and Guidot 2012).

Even more recently, researchers have identified an association between underlying AUD and acute respiratory distress syndrome (ARDS). ARDS is a severe form of acute lung injury that occurs as a complication of diverse insults, including sepsis, massive aspiration, and trauma; it has a mortality rate of 30 percent to 50 percent, even with state-of-the-art modern medical care in an intensive care unit (Villar et al. 2011; Wang et al. 2014; Ware 2006; Ware and Matthay 2000). In 1996, a seminal study demonstrated for the first time that AUD independently conferred an approximately twofold increase in risk of developing ARDS (Moss et al. 1996). A subsequent prospective study focusing only on patients with severe sepsis revealed that the relative risk of developing ARDS was closer to fourfold higher in those with an underlying AUD;^1^ this effect was independent of factors such as age, smoking, severity of illness, and nutritional status (Moss et al. 2003). Other investigators have confirmed these associations (Iribarren et al. 2000; Licker et al. 2003; Spies et al. 1996; von Heymann et al. 2002). Taken together, all of these findings indicate that drinking patterns that define AUD are associated with a significantly increased risk of serious lung infections and acute lung injury and thereby contribute to the deaths of tens of thousands of Americans every year, and many more worldwide.

This review first will discuss key aspects of the epidemiology and pathophysiology of AUD and lung health, before focusing more in-depth on lung infections and acute lung injury, which comprise the majority of alcohol-related lung diseases. The article also will briefly review some of the experimental therapies that hold promise for decreasing the enormous morbidity and mortality caused by the “alcoholic lung” in our society.

## Alcohol and the Airways

The potential influence of alcohol consumption on airway health and disease has been documented for a long time. Chronic alcohol ingestion constantly subjects the drinker’s airways to high concentrations of alcohol vapor, as best evidenced by the use of alcohol breath tests (i.e., Breathalyzer). The volatile nature of alcohol is exploited in this common field sobriety test, which is reliably used as a surrogate to quantify blood alcohol concentrations. Interestingly, the alcohol vapor found in the airways is not caused by inhalation but is a result of the ready diffusion of alcohol from the airway blood supply across the airway epithelium and into the airways themselves (George et al. 1996). This process explains why alcohol vapor in the breath may be used to determine blood alcohol concentration. The alcohol then is deposited and metabolized in the airways. This process leads to the formation of reactive aldehydes (e.g., acetaldehyde), which in turn can interact and form harmful adducts with proteins and DNA (Sapkota and Wyatt 2015). The formation of these adducts may disrupt normal cellular functions, induce inflammation, and impair healing. Taken together, these findings demonstrate that the airways—including the oral cavity and extending all the way to the alveolar space—are subjected to high concentrations of alcohol and its deleterious metabolites during intoxication.

Within the upper airways, chronic alcohol consumption leads to several alterations. First, chronic heavy drinking often is associated with poor tooth development and arrangement (i.e., dentition) as well as poor oral hygiene, and although these usually are attributed to poor nutritional and lifestyle choices, clinical studies have established that they also result, to some extent, from the direct effects of alcohol exposure on the upper airway. Specifically, alcohol decreases saliva production in the salivary glands located in front of the ears (i.e., the parotid glands) (Dutta et al. 1989), thereby eliminating an important mucosal defense within the oral cavity. As a result, heavy drinkers are susceptible to dental caries and gingivitis (Friedlander et al. 2003), conditions that may be exacerbated by concurrent tobacco use, which is common in people with AUD. This alters the microenvironment of the mouth, making it more susceptible to colonization with certain bacteria, including gram-negative bacilli (Fuxench-Lopez and Ramirez-Ronda 1978). Moreover, acute alcohol intoxication and the resulting decrease in the level of consciousness promotes aspiration of oral secretions into the lower airways because of diminished gag and upper-airway reflexes that would normally protect against this phenomenon. These modifications in the upper airways seem to contribute to the increased risk of lung infections, including those caused by more virulent gram-negative organisms, in chronic heavy drinkers.

This risk further is exacerbated by the negative effects of chronic alcohol ingestion on the lower airways. In particular, animal models have established that chronic excessive alcohol ingestion causes dysfunction of the mucociliary apparatus, an important host defense mechanism responsible for clearing harmful pathogens and mucus from the lower airways (Happel and Nelson 2005). An early experimental study in sheep investigating the effects of alcohol on ciliary beat frequency (CBF) demonstrated a dose-dependent effect, such that low alcohol concentrations actually stimulated CBF, whereas high concentrations impaired it (Maurer and Liebman 1988). Later mechanistic studies found that whereas short-term alcohol exposure causes a transient increase in CBF, chronic exposure desensitizes the cilia so that they cannot respond to stimulation (Wyatt et al. 2004). Alcohol-induced failure of the mucociliary system could interfere with the clearance of pathogens from the airways and thereby may contribute to the increased risk of pulmonary infections in people with chronic heavy alcohol use (Sisson 2007).

Although alcohol’s influences on upper and lower airway host defenses collectively are harmful, its role in causing specific diseases, such as asthma, within the conducting airways is less clear (Ayres 1987), despite some interesting historical references. For example, some documentation in Egyptian papyri dating back to about 2000 b.c.e. suggests the use of alcohol in the treatment of asthma (Leake 1952), although one cannot be certain of the accuracy of the asthma diagnosis in these ancient writings. More than 1,000 years later, Hippocrates—who is regarded as the father of Western medicine—noted that wine has a variety of medicinal uses and is specifically beneficial for reducing sputum production (Lucia 1963); again, however, it is not clear if he was referring to asthma as we currently define the syndrome. Much more recently, Salter (1863) described the successful use of alcohol to treat three patients with intractable asthma who had failed all other treatments. In contrast, more modern epidemiological data suggest that chronic heavy drinking is associated with increased odds of all-cause mortality and hospitalization among patients with asthma, although a direct link between asthma control and alcohol use was not investigated (Sumino et al. 2014).

To supplement the various anecdotal reports of using alcohol in the treatment of airway diseases, early mechanistic investigations demonstrated that alcohol itself seems to have bronchodilating properties in asthmatics. However, the effects differed depending on the alcohol concentration used as well as on the route of administration (i.e., intravenous versus oral) (Ayres and Clark 1983*b*; Ayres et al. 1982; Brown 1947; Herxheimer and Stresemann 1963). Moreover, these observations directly conflict with findings that many asthmatics actually report exacerbations of their disease after alcohol ingestion (Ayres and Clark 1983*a*; Breslin et al. 1973; Vally et al. 2000). In an attempt to explain some of these discrepancies, Breslin and colleagues (1973) compared the effects of exposure to different types of alcohol in a clinical study. These analyses found that whereas pure alcohol did not appear to induce bronchial reactivity, some alcoholic beverages worsened asthma symptoms. These findings were the first to suggest that the nonalcohol components and additives of alcoholic beverages may be responsible for inducing asthma, rather than alcohol itself. Similar findings were seen in later studies that examined the effects of red wine in asthma (Dahl et al. 1986; Vally et al. 2000). However, researchers have not yet been able to determine conclusively if alcohol ingestion has any clinically significant effects on asthma. For example, Bouchard and colleagues (2012) showed that alcohol exposure triggered asthma-like pulmonary inflammation in an allergen-sensitized mouse model. The alcohol-exposed mice exhibited increased numbers of certain inflammatory cells (i.e., eosinophils) in fluid obtained from the lungs (i.e., bronchoalveolar lavage fluid), increased production of the main component of mucus (i.e., mucin), and constriction of the small airways (i.e., decreased bronchiole patency). These effects were not seen in mice that were exposed to alcohol but were not allergen sensitized, suggesting that alcohol can be an important trigger for airway reactivity in the context of an underlying allergic component. In contrast, Oldenburg and colleagues (2012) demonstrated that alcohol actually reduced airway hyperresponsiveness and airway inflammation in a mouse model of allergic asthma.

One potential explanation for the disparate findings in the literature regarding alcohol’s role in airway disease is that some forms (i.e., phenotypes) of asthma may be more sensitive to the effects of alcohol than others. One subtype of asthma called aspirin-sensitive asthma or aspirin-exacerbated respiratory disease represents less than 10 percent of all asthma cases (Stevenson and Szczeklik 2006) but accounts for a disproportionately high number of severe asthma cases and can be extremely difficult to diagnose and treat (Berges-Gimeno et al. 2002). Interestingly, alcohol-induced respiratory symptoms are more common in patients with aspirin-exacerbated respiratory disease than in aspirin-tolerant asthmatics (Cardet et al. 2014). These findings suggest that the potential irritant versus bronchodilator effects of alcohol may vary by disease subtype; however, further investigation is necessary to validate these observations.

## Alcohol and Acute Lung Injury

ARDS is a severe form of lung injury characterized by fluid accumulation in the lung that is not related to heart problems (i.e., noncardiogenic pulmonary edema) as well as by flooding of the alveolar airspaces with protein-like (i.e., proteinaceous) fluid (Ware 2006; Ware and Matthay 2000). ARDS develops in response to inflammatory stresses, including sepsis, trauma, gastric aspiration, pneumonia, and massive blood transfusions (Ware and Matthay 2000). Originally described by Ashbaugh and colleagues (1967), ARDS is characterized by alveolar epithelial and endothelial barrier disruption, dysfunction of the lipoprotein complex (i.e., surfactant) coating the lung surfaces, and intense inflammation. Together, these alterations profoundly disrupt gas exchange and cause severe respiratory failure. Although much has been learned about the underlying pathophysiology of this syndrome over the past four decades, treatment of ARDS remains essentially supportive, and despite aggressive treatment in intensive care units and mechanical ventilation, the mortality rate for ARDS remains unacceptably high at 30 percent to 50 percent (Arcasoy et al. 2005; Villar et al. 2011; Wang et al. 2014; Ware and Matthay 2000).

The association between alcohol abuse and acute lung injury only has been identified within the past 20 years, when Moss and colleagues (1996) analyzed a patient database at the University of Colorado and reported that patients who were admitted to the hospital with a critical illness and who had underlying alcohol abuse were at about twofold-increased risk for developing ARDS. This original study was limited by the uncertain accuracy regarding the diagnosis of an underlying AUD in the database. However, a subsequent prospective study of 220 patients with septic shock, in whom a more precise diagnosis of an AUD was established using the Short Michigan Alcohol Screening Test, determined that the incidence of ARDS in patients with AUD was 70 percent (46 of 66), compared with 31 percent (47 of 154) in patients without AUD (*P* < 0.001) (Moss et al. 2003). After controlling for potentially confounding variables, the relative risk of ARDS in alcoholic versus nonalcoholic patients was 3.7:1. Overall, 49 percent (46 of 93) of those patients that developed ARDS had an underlying AUD, consistent with the findings of the earlier study (Moss et al. 1996), in which 51 percent of the patients who developed ARDS were classified as alcoholics. If these findings are extrapolated to the population at large, then alcohol abuse contributes to the development of acute lung injury in tens of thousands of patients in the United States each year.

## Alcohol-Related Mechanisms of Lung Injury

### Disruption of the Epithelial Barrier

The recognition that excessive chronic alcohol ingestion has such a dramatic and independent effect on the risk of acute lung injury prompted a search for the underlying mechanisms. Because one of the cardinal features of ARDS is disruption of the alveolar epithelial barrier that regulates the fluid content of the airspace, this was a logical target for investigation. Maintaining the fluid balance of the alveolar space is critical for normal gas exchange. Acute lung injury involves the rapid development of noncardiogenic pulmonary edema, and patients with impaired alveolar epithelial fluid clearance are three times more likely to die from ARDS than patients with a maximal ability to clear lung fluid (Ware and Matthay 2001). Although the fluid balance in the lungs is regulated by the concerted actions of both epithelial and endothelial barriers (Mehta et al. 2004), it is the alveolar epithelium which primarily prevents protein and fluid flow into airspaces (Mutlu and Sznajder 2005). A pathological hallmark of ARDS is heterogeneous damage of the alveolar epithelium, with complete loss of the epithelial surface in some areas, whereas other alveoli remain relatively intact. Therefore, at a cellular level the extent of the alveolar epithelial damage may not be as widespread or as uniform as chest X-rays may suggest, and preservation and repair of the alveolar epithelium are key to survival.

**Figure 1:**
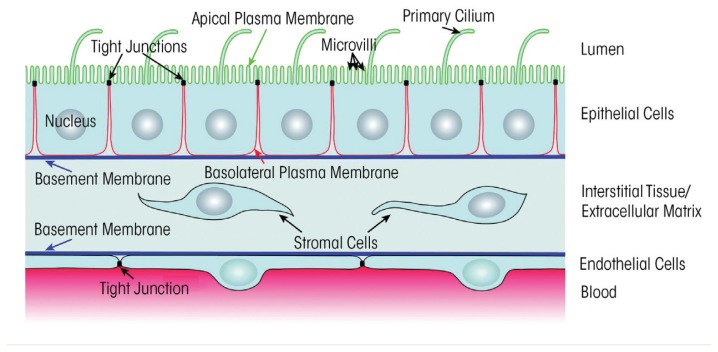
A representation of the alveolar space. In patients with alcohol use disorder (AUD), alterations occur in the tight junctions between alveolar epithelial cells so that protein-rich fluid from the blood can more easily traverse the interstitial tissue and enter the lumen of the alveoli that is normally dry. These and other changes in alveolar epithelial cells predispose people with AUD to developing acute respiratory distress syndrome (ARDS) that is characterized by pulmonary edema.

In experimental animal models, chronic alcohol ingestion for as little as 6 weeks renders the lung susceptible to acute edematous injury (Holguin et al. 1998; Velasquez et al. 2002). In these same models, chronic alcohol ingestion produces a lasting defect in the ability of the alveolar epithelium to form and/or maintain a tight physical barrier; specifically, primary alveolar epithelial cells isolated from alcohol-fed animals form relatively leakier monolayers in culture, even if there is no alcohol in the culture medium (Guidot et al. 2000). In addition, the permeability of the alveolar epithelium to large proteins in vivo is increased approximately fivefold in the alcohol-fed rats (Guidot et al. 2000). The mechanisms by which alcohol impairs the alveolar epithelial barrier are still being investigated. Animal models suggest that chronic alcohol ingestion interferes with the expression and formation of tight junction complexes within the alveolar epithelium (see figure 1) (Fernandez et al. 2007). Tight junctions are closely associated areas of two cells where the membranes of the cells join together; they are critically necessary to form an impermeable barrier that can limit the passage of even very small molecules across cell layers (Koval 2013; Mitic et al. 2000; Schneeberger and Lynch 1992). Only a few studies of alcohol’s effects on the alveolar epithelium have been conducted in humans. The findings indicate that people with AUD have impaired alveolar-capillary permeability at baseline and develop more pulmonary edema in the setting of ARDS compared with people without AUD (Berkowitz et al. 2009; Burnham et al. 2009).

The experimental evidence that alcohol can cause a profound defect in the physical barrier of the alveolar epithelium led to the question of why alcohol abuse alone, in the absence of an acute stress such as sepsis, does not cause pulmonary edema. Additional studies revealed that alcohol causes a concurrent, and perhaps compensatory, increase in salt and water transport across the epithelium. This transport is mediated by specific epithelial sodium channels located in the apical membrane and by protein pumps (i.e., Na/K-ATPase complexes) in the basolateral membrane of the epithelial cells. The expression and function of both the Na/K-ATPase complexes and epithelial sodium channels are increased in the alveolar epithelium of alcohol-fed animals (Guidot et al. 2000; Otis et al. 2008). Thus, as long as there are no additional stresses, the alcoholic lung seems to be able to limit edema formation by upregulating salt and water transport across the epithelium, thereby compensating for the marked increase in the leakage of fluid between cells (i.e., paracellular leakage) into the airways. In the presence of an acute inflammatory stress, such as sepsis or aspiration, however, the paracellular leak increases dramatically, and the alveoli flood with proteinaceous edema fluid that overwhelms the already upregulated transepithelial pumping mechanisms. This scenario is supported by findings in laboratory animals that even at baseline, the lungs of alcohol-exposed animals are unable to clear a salt and water challenge as efficiently, despite the compensatory increase in epithelial sodium channel and Na/K-ATPase function, reflecting the severe permeability defect in the paracellular barrier mechanisms (Guidot et al. 2000).

### Reduced Antioxidant Levels

Another fundamental mechanism that appears to drive many of the pathophysiological manifestations of the alcoholic lung phenotype is a severe depletion of glutathione stores within the alveolar space. Glutathione is the primary thiol antioxidant found in the alveoli; it serves an essential function in reactions catalyzed by the enzyme glutathione peroxidase, which clears harmful hydrogen peroxide and lipid hydroperoxides that readily form in the oxidizing environment of the lung. In both experimental animal models and humans, chronic alcohol ingestion causes a profound decrease of up to 80 percent to 90 percent in alveolar glutathione levels (Holguin et al. 1998; Moss et al. 2000). This glutathione depletion cannot be explained by dietary deficiency or smoking because it also occurs in experimental animals with an otherwise sufficient diet (Holguin et al. 1998); moreover, otherwise healthy smokers actually have increased glutathione levels within their alveolar space (Moss et al. 2000). Further analyses in experimental models found that alcohol-induced glutathione depletion seems to mediate the defects in alveolar epithelial barrier function. For example, when glutathione precursors such as procysteine or S-adenosylmethionine (SAMe) were added to the diet of alcohol-fed animals, the animals’ alveolar epithelial barrier function was restored, and they no longer exhibited increased susceptibility to acute edematous injury compared with control-fed animals (Guidot et al. 2000; Holguin et al. 1998; Velasquez et al. 2002).

Another key function of the alveolar epithelium, namely the synthesis and secretion of surfactant—which is required to maintain alveolar integrity and gas exchange—also is impaired by chronic alcohol ingestion (Holguin et al. 1998). This impairment also is mediated by glutathione deficiency in the cells, and particularly in the mitochondria, and is reversible with dietary procysteine supplementation (Guidot and Brown 2000). Although these animal models provide convincing evidence implicating glutathione depletion as a mediator of alveolar epithelial barrier dysfunction, additional studies in humans are necessary to confirm these findings.

The depletion of glutathione within the alveolar space of people with AUD explains many of the alcohol-related defects in the function of the alveolar epithelium as well as in the function of immune cells called macrophages (which will be discussed in the next section). But how does alcohol lower glutathione so profoundly? Glutathione levels are affected by oxidative stress and inflammation; however, lungs of alcohol-exposed animals show no gross evidence of inflammation or injury at baseline, and otherwise healthy alcoholics likewise have no indication of lung inflammation or oxidative stress. Without evidence of an oxidant assault on the otherwise healthy alcoholic lung, the question remains why there is such overwhelming glutathione depletion. An intriguing answer comes from recent studies showing that, at least in experimental models, chronic alcohol ingestion inhibits the expression and function of a protein called Nrf2. This protein is a master transcription factor that binds to the antioxidant response element (ARE) in the regulatory (i.e., promoter) region of hundreds of antioxidant and immune-response genes (Jensen et al. 2013).

The alcohol-induced inhibition of Nrf2–ARE signaling is mediated at least in part by zinc. Specifically, Nrf2 function depends on adequate zinc levels, and alcohol interferes with the transporter molecules that mediate zinc absorption from the diet as well as its transport into the alveolar space (Joshi et al. 2009). Consistent with this proposed mechanism, dietary supplementation with zinc restores Nrf2 binding to the AREs, preserves the glutathione pool within the alveolar space, and enhances alveolar epithelial barrier function in alcohol-fed rats (Joshi et al. 2009; Mehta et al. 2011; for more information on the role of zinc deficiency and zinc supplementation see the article by Barve and colleagues). These observations suggest that chronic alcohol ingestion lowers antioxidant defenses by interfering with Nrf2-dependent production of antioxidants, including glutathione, and that this may be one mechanism by which alcohol increases the lung’s susceptibility to oxidant stress and, consequently, such conditions as sepsis, pneumonia, or infections resulting from aspiration.

### Other Mechanisms

The pathophysiological mechanisms discussed thus far undoubtedly are just components of a highly complex network of alcohol-induced cellular perturbations. Experimental animal models have elucidated other key aspects of the alcoholic lung, including the role of two signaling molecules, transforming growth factor beta1 (TGFβ1) and granulocyte/macrophage colony-stimulating factor (GM-CSF), both of which help control the growth and function of immune cells, such as macrophages. In healthy people there is relatively little TGFβ1 in the adult lung; instead, alveolar epithelial integrity and the function of alveolar macrophages are under the influence of GM-CSF. Chronic alcohol ingestion increases the expression of TGFβ1 in the lung (Bechara et al. 2004), and its activation during an acute stress situation, such as alcohol-induced transfer of toxic bacterial products from the intestine into the blood (i.e., endotoxemia), seems to further disrupt the already dysfunctional epithelial barrier function (Bechara et al. 2004). Moreover, chronic alcohol ingestion dampens the expression of GM-CSF receptors in alveolar epithelial cells and macrophages (Joshi et al. 2006). This relative imbalance in TGFβ1 and GM-CSF signaling in the alcoholic lung has important implications in the human lung epithelium, and critically ill patients with relatively higher ratios of TGFβ1 to GM-CSF in their alveolar space seem to have a higher mortality (Overgaard et al. 2015). The role of these two signaling molecules is supported by the observation that treatment with recombinant GM-CSF can rapidly restore alveolar epithelial function in alcohol-fed rats, both in vivo and in vitro (Pelaez et al. 2004).

**Figure 2:**
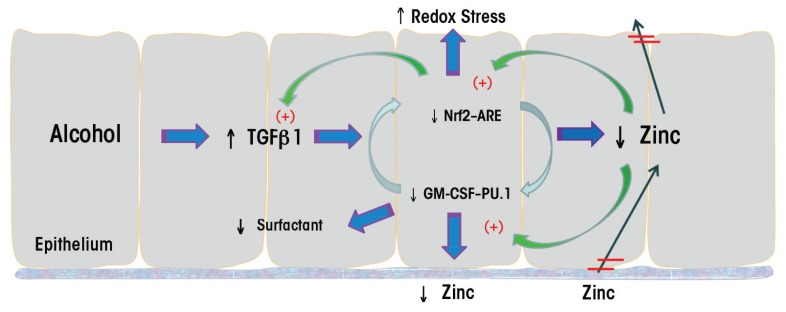
Hypothetical scheme of alcohol’s effects on the alveolar epithelium. Alcohol induces aberrant transforming growth factor beta1 (TGFβ1) expression in the alveolar epithelium and thereby dampens signaling through the granulocyte/macrophage colony-stimulating factor (GM-CSF)–PU.1 and Nrf2–antioxidant responsive element (ARE) signaling pathways. As a consequence, the expression and function of transporters that regulate zinc import and export across the epithelium are disrupted, further inhibiting these zinc-dependent pathways and exacerbating TGFβ1 expression. This results in an increase in redox stress, reduced surfactant levels, and damage to the tight junctions between cells, with severe ramifications for epithelial (and macrophage) function.

Alcohol’s effects on TGFβ1 also interface with its effects on antioxidant levels. Thus, in animal models the alcohol-induced increase in TGFβ1 decreased Nrf2 expression and function and interfered with the resolution of acute lung injury induced by bleomycin; this effect of alcohol could be mitigated by dietary supplementation with SAMe (Sueblinvong et al. 2014; for more information on the role of SAMe supplementation, see the article by Barve and colleagues). Interestingly, Nrf2 also regulates the expression of PU.1, a master transcription factor that mediates GM-CSF–dependent signaling (Staitieh et al. 2015). Accordingly, alcohol-induced reduction of Nrf2 also inhibits binding of PU.1 to its nuclear targets, which can be improved by zinc treatment (Mehta et al. 2011). Thus, alcohol impairs epithelial barrier function in the lung through a complex set of mechanisms with several cycles and feedback mechanisms (see figure 2); however, future studies will almost certainly elucidate further details.

## Alcohol, Alveolar Macrophages, and Pneumonia

Lung infections are major causes of morbidity and mortality worldwide. Data from the Centers for Disease Control and Prevention consistently show that pneumonia is one of the top 10 causes of death in the United States and remains the leading cause of death from an infection (Murphy et al. 2013). Alcoholism has been linked to pulmonary infections for over 200 years (Mehta and Guidot 2012). Additionally, recent studies have demonstrated that people who abuse alcohol are not only more likely to develop pneumonia, but also are susceptible to more severe forms of the disease, are more likely to experience complications, and require greater use of resources. A prospective study by Adamuz and colleagues (2011) examined features associated with increased use of health care services and risk for readmission in patients discharged with pneumonia. Interestingly, the only independent risk factor associated with increased health care utilization after discharge was alcohol abuse. Similarly, Chalmers and colleagues (2009) demonstrated in a multivariate regression model that alcohol abuse was among independent risk factors that were significantly associated with the development of certain complications (i.e., complicated para-pneumonic effusion or empyema) in patients with community-acquired pneumonia.

Another experimental study using a pulmonary infection model of respiratory syncytial virus in mice found that chronic alcohol ingestion caused not only more severe infections, but also influenced the levels of various signaling molecules (i.e., cytokines), inducing a more robust proinflammatory cytokine profile (Jerrells et al. 2007). In this particular study, pulmonary inflammation in alcohol-exposed mice persisted for more than 7 days after infection, compared with 3 to 5 days in the control animals. Moreover, some alcohol-exposed mice showed severe inflammation with hemorrhage and edema. These results corroborate findings that infection in the setting of alcohol exposure increases the risk of complications such as ARDS. Similarly, other studies showed that people with AUD not only are more prone to develop community-acquired pneumonia, but are likely to suffer from infections that portend a worse prognosis and are more likely to be caused by virulent microorganisms that are more challenging to treat (Chen et al. 2001; Fernandez-Sola et al. 1995).

The mechanisms by which chronic and excessive alcohol consumption increases susceptibility to pneumonia are multifactorial. In addition to the already-discussed alterations in bacterial colonization and impaired host defenses in the upper and lower airways, increasing evidence suggests that chronic alcohol ingestion negatively impacts the immune functions of alveolar macrophages in a manner that is similar to its effects on epithelial barrier function. The alveolar macrophage is the primary immune cell in the alveolar space and is responsible for maintaining homeostasis of the lower airways through phagocytosis of pathogens and removal of debris. Animal studies have shown that chronic alcohol exposure causes significant alveolar macrophage dysfunction, leaving these normally active immune cells poorly equipped to phagocytose or kill invading organisms (Brown et al. 2009; Joshi et al. 2009). Alveolar macrophages in alcohol-exposed animals also exhibit decreased production of important chemokines and mediators, which impairs their ability to recruit other cell types, namely neutrophils, during times of stress and infection (Happel et al. 2004). Although the majority of data focuses on the effects of chronic alcohol ingestion, experimental evidence further suggests that even acute exposure has similar detrimental effects on alveolar macrophage immune function, although these defects readily resolve (Libon et al. 1993). Taken together, these alcohol-mediated defects in alveolar macrophage function contribute to increased vulnerability to pulmonary infections.

### Mechanisms of Alcohol’s Effects on Alveolar Macrophages

The precise mechanisms by which alcohol impairs alveolar macrophage immune function have yet to be elucidated; however, several observations indicate that the macrophages are subjected to an altered environment characterized by oxidative stress and zinc deficiency. Both clinical and experimental studies have detected increased oxidative stress in the alveolar space after alcohol exposure (Moss et al. 2000; Velasquez et al. 2002). The exact mechanisms responsible for inducing this redox imbalance remain uncertain, but several explanations have been put forth. An experimental rat model of chronic alcohol ingestion identified perturbations in lipid metabolism analogous to what is seen in alcohol-induced fatty liver (Romero et al. 2014). These alterations included suppression of genes responsible for fatty acid metabolism in the lungs of the alcohol-exposed rats, which caused accumulation of triglycerides and free fatty acids in the distal airspaces and resulted in immune dysfunction of the alveolar macrophages. In another model using mice, Yeligar and colleagues (2012) demonstrated that alcohol induced oxidative stress through the upregulation of specific enzymes called NADPH oxidases, which are an important source of oxidants called reactive oxygen species in alveolar macrophages. A similar pattern of NADPH upregulation existed in human alveolar macrophages isolated from people with AUD. Restoring the redox balance in the lung could reverse many of these alcohol-induced defects and improve alveolar macrophage immune function (Brown et al. 2007; Yeligar et al. 2014).

Also, as noted above, chronic alcohol ingestion interferes with Nrf2 signaling in alveolar macrophages (Mehta et al. 2011), thereby disrupting the expression of hundreds of genes that are crucial to combatting oxidative stress. Although the precise role of alcohol-mediated inhibition of the Nrf2–ARE pathway in mediating oxidative stress has not been completely clarified, this pathway represents a strategic target to direct future therapies.

Another important player in alveolar macrophage-mediated immunity is zinc. In experimental models, alveolar macrophages from alcohol-fed animals exhibit zinc deficiency in the fluid of the epithelial lining and have decreased intracellular zinc levels compared with alveolar macrophages from control-fed animals (Joshi et al. 2009). These findings have been confirmed in alveolar macrophages collected from otherwise-healthy people with underlying AUD, even though these individuals had normal serum levels of zinc (Mehta et al. 2013). Zinc is important for diverse immune functions, and its severe deficiency within the alveolar space may be one mechanism by which alcohol impairs innate immune functions within the lung. This role is further supported by findings that restoration of zinc bioavailability in the alveolar space also restores the phagocytic capacity of alveolar macrophages (Joshi et al. 2009).

As discussed previously, alcohol not only alters the environment of the alveolar space but also directly affects GM-CSF signaling, which regulates the maturation, terminal differentiation, and function of alveolar macrophages. Chronic alcohol ingestion downregulates the expression of GM-CSF receptors on the cell surface of the alveolar macrophages, thereby impairing their immune function (Joshi et al. 2005). Experimental models demonstrate that restoration of GM-CSF signaling reverses this alcohol-induced dysfunction (Joshi et al. 2005), suggesting that this might be a potential therapeutic approach. Also, as mentioned earlier, recent evidence suggests that interactions exist between Nrf2 and the GM-CSF pathway, with Nrf2 regulating the expression and activity of the transcription factor PU.1, which controls GM-CSF expression (Staitieh et al. 2015). Understanding the complex interplay between all of these systems in the alcoholic lung will become exceedingly important in the search for new and effective treatments. For example, zinc supplementation in experimental models of chronic alcohol ingestion improves redox balance, enhances Nrf2 binding in the nucleus, corrects alveolar macrophage immune dysfunction, and restores GM-CSF receptor expression and signaling, suggesting that one target can interact with several implicated pathways (Joshi et al. 2009; Mehta et al. 2011).

Overall, these alterations in host defense and immune dysfunction explain how chronic excessive alcohol ingestion predisposes to pulmonary infection. It is important to realize, however, that the effects of alcohol on alveolar macrophage innate immune function are just one facet of the complex pathophysiology of alcohol and the lung’s immune system. Alcohol also impairs neutrophil migration to the infected lung, and abnormalities in this and other components of the adaptive immune response clearly are involved but are beyond the scope of this brief review.

## Potential Therapeutic Strategies for the Alcoholic Lung

Currently there are no specific therapies that can modify the alcoholic lung in the clinical setting. Clearly, as with all alcohol-related health issues, the ideal treatment would be abstinence in people with underlying AUD and/or a safe level of consumption in people who choose to drink for social reasons. However, this ideal will be impossible to achieve in any meaningful timeframe and it therefore is critical to identify, test, and validate therapeutic strategies that can limit the morbidity and mortality of alcohol-related diseases, including acute lung injury and pneumonia.

For identifying candidate approaches, it is important to recognize that a large percentage of people with AUD are otherwise healthy and can be identified by relatively simple health-screening questionnaires well before they develop serious organ dysfunction (de Oliveira et al. 2014; Spithoff and Kahan 2015). Also, many people with AUD seek treatment, and structured alcohol treatment programs offer an opportunity to initiate adjunctive therapies designed to enhance lung health. The experimental results reviewed in this article provide some suggestions for promising approaches that could be used in such settings. For example, as discussed previously, clinical studies have shown that even otherwise-healthy people with AUD have glutathione and zinc deficiency within the alveolar space (Mehta et al. 2013; Moss et al. 2000). Moreover, animal studies found that dietary supplementation with zinc and/or a glutathione precursor such as SAMe can enhance lung health even in the context of chronic alcohol ingestion (Guidot et al. 2000; Holguin et al. 1998; Joshi et al. 2009; Mehta et al. 2011; Velasquez et al. 2002; also see the article by Barve and colleagues). Accordingly, researchers at the Atlanta VA Medical Center initiated a randomized, placebo-controlled trial of dietary zinc and/or SAMe in otherwise-healthy individuals with AUD enrolled in the center’s Substance Abuse Treatment Program (available at: clinicaltrials.gov, trial NCT01899521). This trial currently is in progress with the goal of determining whether these supplements, alone or in combination, can enhance glutathione and zinc bioavailability in the alveolar space and improve alveolar macrophage immune function.

Another potential therapeutic target is Nrf2, which can be activated by plant-derived compounds (i.e., phytochemicals), such as sulforaphane (Hybertson et al. 2011; Jensen et al. 2013). One clinical study (Burnham et al. 2012) evaluating the effects of 7-day treatment with the Nrf2 activator Protandim® in patients with AUD did not identify any significant improvement in glutathione levels or epithelial function. However, it is possible that combination therapy with an Nrf2 activator plus zinc and/or SAMe may be more effective than zinc and/or SAMe alone, and clinical trials in the near future hopefully will be able to answer that question.

The goal of these treatments clearly would not be to make it safe(r) to consume excessive amounts of alcohol. However, just as clinicians try to mitigate the health effects of metabolic syndrome in obese patients using medications that target diabetes, hypertension, or dyslipidemia while the patients struggle with weight loss, it is imperative to decrease the risk of pneumonia, acute lung injury, and other life-threatening complications while people with AUD work to achieve abstinence. There also may be some concerns about alcoholic patients’ compliance with chronic oral treatments, such as zinc and SAMe supplements. However, many patients with AUD seek care for their addiction precisely because they are motivated to become or remain healthy and, consequently, are likely to adhere to their treatment regimen. Moreover, inadequate adherence to medical regimens is not a concern unique to this patient population but occurs in patients with many chronic medical conditions; examples include the low use of continuous positive airway pressure therapy for obstructive sleep apnea (Russell 2014) and poor adherence with anti-diabetic medications in adults with type 2 diabetes (Sapkota et al. 2015). Even if patients seeking treatment for AUD have equally low adherence rates, tens of thousands of individuals could benefit from these relatively simple and inexpensive treatments every year in the United States alone. Researchers and clinicians are just beginning to scratch the surface of this challenging problem, but the rapid pace of experimental and clinical research in the past two decades offers hope that in the relatively near future the devastating effects of AUD on lung health can be ameliorated.

## Footnotes

1 The diagnosis of an AUD was based on the Short Michigan Alcohol Survey Test (Selzer et al. 1975).

## Glossary

Adduct: A product of a direct addition of two or more distinct molecules, resulting in a single reaction product
Alveolar: Pertaining to the *alveoli*
Alveoli: The small sac-like structures at the end of the airways where gas exchange occurs
Antioxidant: A substance (e.g., glutathione and vitamins A and E) that inhibits oxidation, serving as a defense against harmful free radicals and *oxidative stress*
Aspiration: Entry of material (e.g., food, drink, saliva) from the digestive tract into the airways
Bronchoalveolar Lavage: Procedure where fluid is injected through a tube into a small airway of the lung and then collected and tested for the presence of bacteria and other compounds; the procedure is used to diagnose infections and other lung diseases
Bronchodilating: Causing widening of the airways
Cilia: Fine, hair-like structures on the surface of *epithelial cells* lining the airways; their coordinated movement helps clear microorganisms and other particles as well as mucus from the airways
Edema: Abnormal accumulation of fluid in the tissues and body cavities; typically causes swelling of the tissues
Empyema: Collection of pus in a cavity of the body; if pus accumulation occurs in the pleural cavity, it is considered a subtype of *parapneumonic effusion*
Endothelial Cell: Type of cell lining the interior of body cavities and blood vessels; endothelial cells control the passage of materials and the transit of white blood cells into and out of the bloodstream
Epithelial Cell: Type of cell lining the tissues exposed to the outside of the body, such as the skin, but also the lining of the intestine and airways
Gingivitis: A common gum disease that causes irritation and inflammation of the gums, characterized by redness and swelling of the gums
Granulocyte/Macrophage Colony-Stimulating Factor (GM-CSF): Cytokine released by various white blood cells and lung *epithelial cells* that stimulates the growth of additional white blood cells; it is produced as part of the immune response so that activation of a small number of immune cells, particularly macrophages, leads to a rapid increase in the number of these cells
Leukopenia: Abnormal reduction in the number of white blood cells
Mitochondria: Structures within cells that generate most of cell’s energy through the production of adenosine triphosphate (ATP)
Mucociliary: Pertaining to the mucus and the *cilia* of the *epithelial cells* lining the airways
Mucociliary Apparatus: The layer of cells lining the surface of the respiratory tract that are covered by a thin layer of mucus and which carry *cilia*, whose rhythmic beating helps clear the airways
Oxidative Stress: An imbalance between oxidants (e.g., free radicals) and *antioxidants* that can lead to excessive oxidation and cell damage
Parapneumonic Effusion: Type of fluid accumulation between the two membranes surrounding the lungs (i.e., in the pleural cavity) that results from pneumonia and other lung disorders
Permeability: Capacity of membranes (e.g., the walls of the blood vessels) to allow the passage of fluids, small molecules, or even whole cells from one side of the membrane to the other
Phagocytosis: Process by which certain cells (phagocytes) engulf foreign particles in special small vesicles where they can be destroyed by enzymes
Promoter: A DNA segment located at the start of a gene’s coding sequence that provides a binding site for the enzymes that initiate transcription
Reactive Oxygen Species (ROS): Highly reactive oxygen–containing free radicals that are generated during oxidative metabolism. ROS can react with and damage lipids, proteins, and DNA in cells, causing *oxidative stress*. Common ROS include hydrogen peroxide, superoxide radicals, and hydroxyl radicals
Sepsis: Inflammatory response throughout the body in response to an infection; can lead to multiple organ failure and death
Surfactant: Lipoprotein complex formed by *alveolar* cells that helps ensure the lungs’ ability to expand during respiration, regulate the size of the *alveoli,* and prevent fluid accumulation in the lungs; also plays a role in innate immunity of the lungs
Transforming Growth Factor Beta1 (TGFβ1): Cytokine that helps control the growth, proliferation, differentiation, and cell death of different types of immune cells; it thereby helps control the activity of the immune system
Transcription Factor: A protein that regulates gene expression by binding to *promoters* on the DNA near the start of the gene, thereby activating other proteins involved in transcription
